# Editorial: Plant parasitic nematode–host interactions: mechanisms and exploitative management strategies

**DOI:** 10.3389/fpls.2024.1539529

**Published:** 2025-01-09

**Authors:** James Price, Victor Hugo Moura de Souza, Andressa Cristina Zamboni Machado, Mário Massayuki Inomoto

**Affiliations:** ^1^ Cellular & Molecular Sciences, The James Hutton Institute, Dundee, United Kingdom; ^2^ Crop Science Centre – Department of Plant Sciences, University of Cambridge, Cambridge, United Kingdom; ^3^ Department of Nematology, Agronema, Londrina, Brazil; ^4^ Department of Plant Pathology and Nematology – University of São Paulo, Piracicaba, Brazil

**Keywords:** biological control, resistance to pathogens, alternative control of nematodes, effectors, induced resistance

By 2060, the human population on Earth is expected to reach 10 billion people and food production to sustain this population is of great concern. Food security is not only pressurised by abiotic factors associated with a rapidly changing climate but also by biotic, pathogenic threats. In many areas of the world, plant-parasitic nematodes (PPN) are the greatest stress on sustainable crop production. However, PPN parasitise all aspects of plant life and, where left unmanaged, could lead to major ecological collapse. Historically, management of PPN relied on incorporation of harmful, non-specific chemicals into infested soils. Generally, PPN control has moved towards integrated pest management (IPM) approaches. This Research Topic addresses how a better understanding of nematode-host interactions can help further develop management strategies.

Although reliance on nematicidal chemicals should be reduced under an IPM approach, they remain an important part of pest management. Many manufactured products are being removed from international markets due to harmful off-target effects. Consequently, there is urgent need for new nematicidal products which are often being developed with naturally occurring ingredients in agreement with green deals. Having plant origin means that these products can be interpreted by the crop boosting defence pathways for defence against any pathogen, not just PPN, in addition to direct nematicidal effects. This is demonstrated by Degroote et al. in their use of the bio-based ‘Product X’ which was shown to considerably reduce *Meloidogyne incognita* gall formation in tomato plants.

The ongoing boon in availability of -omics data may help shift towards novel bio-based nematode controls such as exogenous application of microRNAs. These technologies offer the opportunity of incredibly targeted control identified through bioinformatics pipelines. Leonetti et al. established the use of a pipeline for identification and creation of cross-kingdom miRNAs, in this case originating from tomatoes and capable of reducing expression of two key *M. incognita* target genes. The use of miRNAs for nematode control is still a developing area and further research will be required on construct delivery in an applied setting.

This Research Topic has shown that PPN-host interactions do not have to be directly coupled and instead understanding host-associated microorganisms could offer fresh management strategies. The impact of plant growth-promoting bacteria (PGPB) on pathogen suppression was reviewed by Habteweld et al.. This review investigates the three-way interactions shared between PPN-host-PGPB and how influencing PGPB communities can assist root-knot nematode management. One demonstrated example of the use of PGPB to promote pathogen resistance is illustrated by Hu et al. who used *Bacillus velezensis* to promote host resistance against *Heterodera glycines*. Here, bacterial stimulation of the jasmonic acid (JA) biosynthesis pathway resulted in increased JA in root tissues supplying a systemic resistance effect. Jia et al. further develop the idea of both influencing plant-associated microorganisms coupled with novel plant-originating product development for nematode suppression. *Aspergillus* sp. promotes coexistence of *Bursaphelenchus xylophilus* and their vector insect in pine trees. However, inhibiting *Aspergillus* populations with chiricanine A decreased nematode populations, increasing host plant survival.

Plant-parasitic nematode population increases due to host availability drive changes in soil microbial communities. Clavero-Camacho et al. explored this further by observing soil and rhizospheric microbiome changes in *Meloidogyne* spp. infected almonds. Under high infection pressures, fungal saprotrophism is altered and there is reduced impact of predatory nematodes and biological control agents. A significant portion of plant-nematology research focusses on the tight evolutionary relationship and interactions between PPN and host, however, PPN have also adapted for life before host invasion. Better understanding these adaptations could help develop biological controls through use of antagonistic microorganisms.

Natural host resistance is playing an increasing role in IPM of plant-parasitic nematodes. Modern breeding of resistant varieties relies on molecular markers for resistance allowing genotyping to reduce populations before often time-consuming phenotyping. Obata et al. utilised a genome-wide association study (GWAS) and next-generation sequencing to develop markers for sweet potato resistance against *M. incognita*. In complex polyploid crop species, e.g., sweetpotato, tools like these rapidly enable the pre-screening of breeding progeny. Understanding how host resistance genes enable a physical response upon detection of PPN can assist knowledge in further resistance gene discovery, pathogen virulence and developing plant protection products. Histopathology techniques used by Gabriel et al. demonstrate that the *Mi-1.2* gene induces both biochemical protection, reducing root invasion, and a hypersensitive response, causing cell death following *M. javanica* infection. However, virulent populations were unimpaired, avoiding host detection in tomatoes bearing resistance gene.

Effector proteins help virulent populations of PPN infect their hosts. Learning about effectors can help identify routes for control by targeting parts of the host-parasite essential for parasitism. Li et al. characterised a novel effector expressed in the oesophageal gland of *Bursaphelenchus. xylophilus* that induces plant cell death and aids migration. Targeting this effector for reduced expression through RNA interference reduced both disease severity and seedling death.

PPN-host exchanges go beyond effector-mediated interactions and as reviewed by Dominguez-Figueroa et al. there are often infection-associated host transcriptomic changes. Although many differentially expressed genes encoding transcription factors (TF) have been identified, relatively few of these TFs have been functionally characterised. However, these TFs can be segregated into two main groups whether they are defence or development-related. Understanding how these TFs are being abused during pathogen attack could help develop innate TF regulatory controls at the detriment of the nematode.

